# Current status and influencing factors of laryngopharyngeal reflux disease in outpatients with laryngeal diseases: key considerations for clinical practice

**DOI:** 10.3389/fmed.2026.1766779

**Published:** 2026-05-18

**Authors:** Longfang Zhang, Yingchuan Wu, Yuanyuan Yang

**Affiliations:** Department of Otolaryngology Head and Neck Surgery/Head and Neck Center, The First affiliated Hospital of Soochow University, Suzhou, JIangsu, China

**Keywords:** clinical, laryngeal, laryngopharyngeal reflux disease, nursing, treatment

## Abstract

**Background:**

Laryngopharyngeal reflux disease (LPRD) is a common comorbidity in outpatients with laryngeal diseases, which seriously affects patients' quality of life and treatment outcomes. This study aimed to explore the current status of LPRD comorbidity in outpatients with laryngeal diseases, identify its independent risk factors, and construct a practical prediction model to provide evidence for clinical prevention and early diagnosis.

**Methods:**

Outpatients with laryngeal diseases treated in our hospital from August 2024 to August 2025 were included. Demographic characteristics, living habits, comorbidities, and examination indicators were collected and compared.

**Results:**

A total of 1,650 outpatients with laryngeal diseases were included. Multivariate Logistic regression analysis confirmed that age ≥65 years, BMI ≥ 25 kg/m^2^, smoking history, preference for strong tea, abnormal LES function, history of laryngeal surgery, and hypertension were risk factors for LPRD (all *P* < 0.001), with abnormal LES function having the highest odds ratio (OR = 2.376, 95% CI: 1.985–2.836). A weighted prediction model was developed based on the β coefficients of these seven factors, with a total score ranging from 0 to 22 points. The optimal diagnostic cutoff was 9.5 points (Youden index = 0.615), yielding a sensitivity of 0.723 and a specificity of 0.892. The area under the curve (AUC) of the combined model was 0.891 (95% CI: 0.870–0.912).

**Conclusion:**

LPRD is prevalent among outpatients with laryngeal diseases and is influenced by multiple risk factors. The prediction model developed based on seven independent risk factors demonstrates good diagnostic performance, offering a practical and reliable tool for early identification of high-risk patients.

## Background

Laryngopharyngeal reflux disease (LPRD) is a cross-disciplinary disorder involving the digestive and laryngopharyngeal systems, characterized by the reflux of gastric contents into the laryngopharynx, airway, and other regions above the upper esophageal sphincter (UES), which induces mucosal injury and a series of clinical symptoms (1, 2). Its core pathophysiological mechanism lies in the impairment of the anti-reflux barrier caused by factors such as lower esophageal sphincter (LES) dysfunction and abnormal gastroesophageal motility. In recent years, with changes in people's lifestyles (e.g., staying up late, increased mental stress) and the accelerated aging of the population, the incidence of LPRD has shown a year-by-year upward trend, with a particularly prominent detection rate among outpatients with laryngeal diseases (3, 4). In clinical practice, LPRD patients often present with non-specific symptoms including laryngopharyngeal foreign body sensation, hoarseness, chronic cough, and pharyngeal itching or pain (5). These symptoms highly overlap with those of primary laryngopharyngeal diseases such as chronic laryngopharyngitis, vocal cord polyps, and laryngeal precancerous lesions, leading to a high rate of clinical misdiagnosis and missed diagnosis (6). Some patients experience recurrent episodes or even progress to severe complications such as vocal cord nodules and laryngeal stenosis due to delayed intervention, which significantly impacts their quality of life and prognosis (7).

The pathogenesis of concurrent LPRD in patients with laryngeal diseases is jointly regulated by multiple factors. Existing clinical studies (8–10) have suggested that age, body mass index (BMI), smoking and drinking history, dietary habits, and other factors may be closely associated with the occurrence of LPRD. For instance, elderly patients are at high risk of LPRD due to decreased elasticity of the lower esophageal sphincter, weakened gastrointestinal motility, and impaired anti-reflux capacity. Obese patients, on the other hand, have increased intra-abdominal pressure, which easily promotes the reflux of gastric contents and elevates the risk of disease onset. In addition, a history of laryngeal surgery may further induce or exacerbate reflux symptoms by altering the anatomical structure of the laryngopharynx and affecting local mucosal sensitivity (11). However, current comprehensive analyses of the impact of these factors on the development of LPRD in patients with laryngeal diseases are still insufficiently systematic, and the independent effects and combined predictive value of each factor require verification with large-sample clinical data. Meanwhile, the lack of a unified and rapid screening tool for the diagnosis of concurrent LPRD in patients with laryngeal diseases in clinical practice results in delayed intervention for some patients with occult reflux, missing the optimal treatment opportunity (12).

Given the high incidence, occult symptoms, and clinical diagnostic and therapeutic complexity of LPRD among outpatients with laryngeal diseases, clarifying the status of concurrent LPRD in this population and systematically analyzing its key influencing factors are of great significance for optimizing clinical diagnosis and treatment strategies, improving diagnostic accuracy, and reducing the incidence of complications (13). Currently, targeted studies on the epidemiological characteristics and risk factors of concurrent LPRD in patients with laryngeal diseases are relatively scarce, especially the lack of large-sample, multi-center empirical data support based on outpatient populations (14). Therefore, this study aims to conduct a retrospective analysis of clinical data from outpatients with laryngeal diseases to clarify the prevalence of concurrent LPRD in this population, screen for its main influencing factors, and provide a scientific basis for clinically constructing accurate risk prediction models and formulating individualized prevention and intervention measures.

## Methods

### Ethical approval

This study was reviewed and approved by the Medical Ethics Committee of the hospital (Ethics Approval No.: 20251172), and all research procedures strictly adhered to the Declaration of Helsinki and relevant medical ethical guidelines (15). As this was a retrospective analysis, data were derived from medical records routinely collected during clinical diagnosis and treatment. All patient identifiers (e.g., name, ID number, contact information) were anonymized during the study. Following ethical committee review, informed consent was waived due to the retrospective nature of the study and the anonymization of patient data. Throughout the research process, strict compliance with medical data privacy protection regulations was maintained to ensure patient information security and prevent the disclosure of any sensitive clinical data.

### Study design

**Figure 1:**
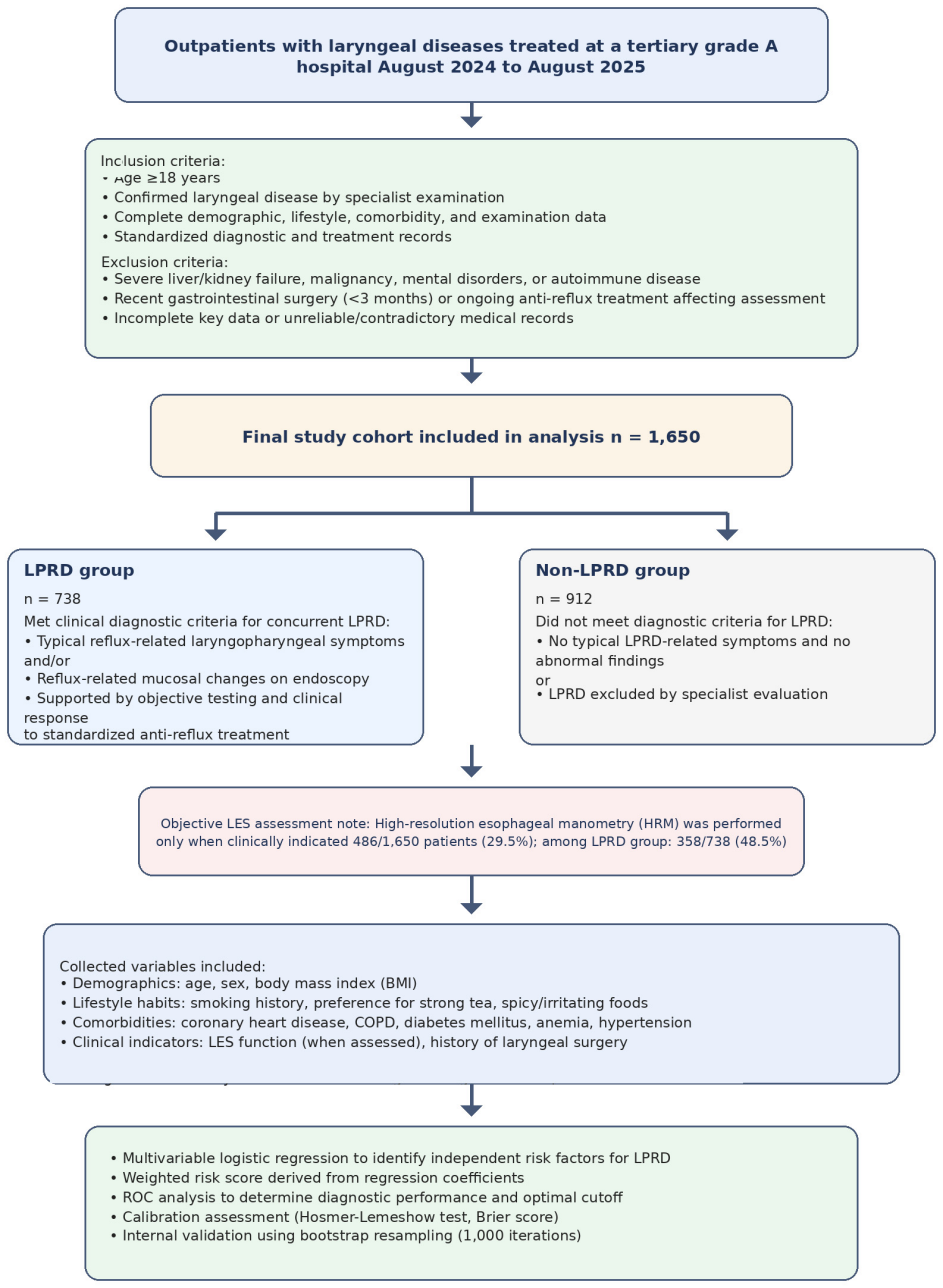
Flowchart of patient screening, grouping, and data analysis.

A retrospective cross-sectional analytical study design was adopted, with participants recruited from outpatients with laryngeal diseases at a tertiary grade A hospital. Patients were divided into the laryngopharyngeal reflux disease (LPRD) group and non-LPRD group based on the presence or absence of concurrent LPRD. By systematically comparing clinical baseline data, lifestyle habits, comorbidities, and results of specialized examinations between the two groups, potential influencing factors for concurrent LPRD in patients with laryngeal diseases were identified. This study design efficiently utilizes existing clinical diagnostic and treatment data, avoiding the long duration and high costs associated with prospective studies. Meanwhile, the case-control grouping logic enables effective exploration of associations between exposure factors and outcomes, providing empirical evidence for the development of targeted clinical prevention and control strategies. A flowchart of the experimental procedure is presented in Figure 1.

### Sample size calculation

Sample size was estimated using formulas for case-control studies, with core parameters set based on previous relevant research: α = 0.05 (two-tailed test) and β = 0.10 (90% statistical power). According to preliminary pilot data and literature reports, the estimated prevalence of LPRD among outpatients with laryngeal diseases was approximately 45%, and the minimum odds ratio (OR) for key exposure factors was set at 1.5. Using PASS 15.0 statistical software, the minimum required sample size was calculated to be 1,320 cases. To account for potential data missing in clinical records and patients who might not meet the inclusion/exclusion criteria, the sample size was expanded by 20% to ensure sufficient statistical power. Ultimately, 1,650 patients were enrolled, including 738 in the LPRD group and 912 in the non-LPRD group.

### Study population

This study included outpatients diagnosed with laryngeal diseases at our hospital between August 2024 and August 2025 as research populations. The inclusion criteria were defined as follows: (1) aged ≥ 18 years with a confirmed diagnosis of laryngeal diseases (including chronic laryngopharyngitis, vocal cord polyps, laryngitis, benign laryngeal hyperplasia, and other related conditions) through specialized examinations in the laryngeal disease outpatient clinic; (2) complete clinical medical records encompassing key information, such as general demographic characteristics, lifestyle habits, comorbidities, and results of relevant laboratory and imaging examinations; and (3) standardized diagnosis and treatment processes, with clear clinical evidence supporting all examination findings and diagnostic conclusions.

Patients were excluded if they met any of the following criteria: (1) comorbidity with diseases that could potentially interfere with study results, including severe liver or kidney failure, malignant tumors, mental disorders, or autoimmune diseases; (2) a history of recent gastrointestinal surgery (within 3 months), long-term treatment with anti-reflux medications without discontinuation, or ongoing treatment that might affect the status of laryngopharyngeal mucosa; (3) incomplete clinical data with missing key exposure factors or outcome indicators, which would preclude valid statistical analysis; or (4) unreliable diagnostic and treatment data or obvious logical contradictions in medical records due to various reasons.

Grouping was conducted in accordance with the clinical diagnostic criteria for LPRD: the LPRD group included participants who met either of the following diagnostic conditions (16, 17)— presence of typical LPRD-related symptoms (e.g., laryngopharyngeal foreign body sensation, persistent hoarseness, chronic irritative cough, pharyngeal itching or pain) accompanied by evidence of lower esophageal sphincter (LES) dysfunction (e.g., relaxation or reduced pressure) on high-resolution esophageal manometry; identification of reflux-related mucosal changes (e.g., laryngopharyngeal mucosal congestion, edema, follicular hyperplasia, vocal cord granuloma) through otolaryngological endoscopy, which was confirmed by corresponding clinical symptoms and significant symptom relief following standardized anti-reflux treatment—while the non-LPRD group consisted of participants without the aforementioned LPRD-related symptoms or abnormal examination findings, or those for whom LPRD was clearly excluded by specialized examinations. Of note, high-resolution esophageal manometry was performed only in patients with clinical indications for LES function assessment, not in all participants (see Results). It must be noted that the Reflux Symptom Index (RSI) and Reflux Finding Score (RFS) are widely used tools for LPRD. However, in this study, the diagnosis of LPRD was based primarily on objective examinations (endoscopy, 24-h pH monitoring) and treatment response, the RSI and RFS were not systematically collected in this study.

### Data collection

A standardized retrospective data collection method was adopted, with a unified data extraction form developed. Trained researchers independently extracted relevant information from electronic and paper medical records in pairs, followed by cross-validation to ensure data consistency. Collected data included: (1) General demographic characteristics: age, gender, body mass index (BMI); (2) Lifestyle habits: smoking history (defined as smoking ≥ 1 cigarette per day for ≥ 1 year), dietary preferences (preference for strong tea, spicy and irritating foods); (3) Comorbidities: coronary heart disease, chronic obstructive pulmonary disease (COPD), diabetes mellitus, anemia, etc.; (4) Relevant clinical indicators: LES motility status (assessed by high-resolution esophageal manometry when clinically indicated), history of laryngeal surgery (previous laryngeal surgeries such as vocal cord polypectomy, laryngoplasty, or laryngeal tumor resection). Missing or contradictory data identified during cross-validation were supplemented or corrected by reviewing original diagnostic records and examination reports; cases with uncorrectable data were excluded.

### Statistical analysis

SPSS 26.0 statistical software was used for data processing and analysis, and GraphPad Prism 9.0 was employed for graphing. Normality testing was performed for continuous variables first: those conforming to a normal distribution were expressed as mean ± standard deviation (x̄±s), with intergroup comparisons using independent samples *t*-test; those not conforming to a normal distribution were expressed as median (interquartile range) [M (Q1, Q3)], with intergroup comparisons using Mann-Whitney *U*-test. Categorical variables were expressed as counts (percentages) [*n* (%)], with intergroup comparisons using χ^2^ test or Fisher's exact test (when the expected frequency < 5). Candidate variables for multivariate logistic regression were prespecified based on clinical relevance, prior literature, and pathophysiological plausibility. Variables with *P* < 0.05 in univariate analysis were considered for inclusion in the multivariate model, which was constructed using forced entry to avoid the potential overfitting and inflated type I error associated with automated stepwise selection. Odds ratios (OR) with 95% confidence intervals (95% CI) were calculated for each independent risk factor. A weighted risk prediction model for LPRD was developed based on the β coefficients from the final multivariate logistic regression model. Receiver operating characteristic (ROC) curves were used to evaluate the diagnostic efficacy of the model and individual influencing factors, with the area under the curve (AUC), sensitivity, specificity, and Youden index calculated to determine the optimal diagnostic threshold of the model. Model calibration was assessed using the Hosmer-Lemeshow goodness-of-fit test, and the Brier score was calculated to evaluate overall model performance. Internal validation was performed using the bootstrap method (1,000 resamples) to assess model stability and reliability. A *P* value < 0.05 was considered statistically significant.

## Results

As shown in Table 1, a total of 1,650 outpatients with laryngeal diseases were included in this study, including 738 cases in the LPRD group and 912 cases in the non-LPRD group. There were statistically significant differences between the two groups in demographic characteristics, living habits, comorbidities, and examination indicators (all *P* < 0.05): the LPRD group had higher average age, higher BMI, and a higher proportion of males compared with the non-LPRD group; in terms of living habits, the proportions of patients with smoking history, preference for strong tea, and spicy/irritating food in the LPRD group were significantly higher than those in the control group; regarding comorbidities, the LPRD group had higher rates of coronary heart disease, COPD, diabetes mellitus, and anemia than the non-LPRD group; in addition, the incidence of abnormal LES function and the proportion of patients with a history of laryngeal surgery in the LPRD group were also significantly higher than those in the non-LPRD group, and all the aforementioned differences were statistically significant. In this cohort, HRM was performed in 486 patients (29.5% of the total cohort) for whom LES function assessment was clinically indicated. Among the LPRD group, 358 patients (48.5%) underwent HRM.

**Table 1:** General data of outpatients with laryngeal diseases and comparison between groups (*n* = 1,650).

Variables	LPRD group (*n* = 738)	Non-LPRD group (*n* = 912)	*t*/χ^2^	*p*
Age (y)	67.25 ± 8.36	61.58 ± 9.12	10.253	< 0.001
BMI (kg/m^2^)	26.89 ± 3.15	22.36 ± 2.87	28.647	< 0.001
Male/female	402/336	438/474	12.385	< 0.001
Smoking history	386 (52.30%)	292 (32.02%)	68.752	< 0.001
Coronary heart disease	185 (25.07%)	168 (18.42%)	11.264	0.001
COPD	152 (20.60%)	135 (14.80%)	10.538	0.001
Diabetes mellitus	162 (21.95%)	156 (17.10%)	8.942	0.003
Anemia	148 (20.05%)	132 (14.47%)	9.876	0.002
Preference for strong tea	326 (44.17%)	215 (23.57%)	89.625	< 0.001
Preference for spicy and irritating food	358 (48.51%)	248 (27.19%)	102.364	< 0.001
Abnormal LES function	412 (55.83%)	185 (20.28%)	268.451	< 0.001
History of laryngeal surgery	126 (17.07%)	68 (7.46%)	45.328	< 0.001

To identify the independent risk factors for LPRD, variables with statistical significance in the univariate analysis and hypertension were included in the multivariate logistic regression model (variable assignment is shown in Table 2, with 0 as the reference category), and the results are presented in Table 3. Ultimately, age ≥ 65 years, BMI ≥ 25 kg/m^2^, smoking history, preference for strong tea, abnormal LES function, history of laryngeal surgery, and hypertension were identified as independent risk factors for LPRD (all *P* < 0.001). Among these, abnormal LES function had the highest odds ratio (OR = 2.376, 95% confidence interval [CI]: 1.985–2.836), indicating that it had the most significant impact on the occurrence of LPRD; followed by BMI ≥ 25 kg/m^2^ (OR = 1.881, 95% CI: 1.536–2.298) and age ≥ 65 years (OR = 1.796, 95% CI: 1.452–2.218). The OR values of the remaining factors ranged between 1.4 and 1.7, suggesting that all these factors could increase the risk of LPRD in patients with laryngeal diseases to varying degrees.

**Table 2:** Variable assignment for multivariate logistic regression of influencing factors for LPRD in outpatients with laryngeal diseases.

Factors	Variables	Assignment
LPRD	Y	Yes = 1, No = 0
Age	X1	≥65y = 1, < 65y = 0
BMI	X2	≥25 kg/m^2^ = 1, < 25 kg/m^2^ = 0
Smoking history	X3	Yes = 1, No = 0
Preference for strong tea	X4	Yes = 1, No = 0
Abnormal LES function	X5	Yes = 1, No = 0
History of laryngeal surgery	X6	Yes = 1, No = 0
Hypertension	X7	Yes = 1, No = 0

**Table 3:** Logistic regression analysis of influencing factors for LPRD in outpatients with laryngeal diseases.

Variables	β	Wald	OR	95%CI	*p*
Age ≥ 65y	0.586	32.654	1.796	1.452–2.218	< 0.001
BMI ≥ 25 kg/m^2^	0.632	41.892	1.881	1.536–2.298	< 0.001
Smoking history	0.425	28.365	1.529	1.278–1.832	< 0.001
Preference for strong tea	0.498	36.528	1.646	1.358–1.992	< 0.001
Abnormal LES function	0.865	78.452	2.376	1.985–2.836	< 0.001
History of laryngeal surgery	0.396	22.158	1.485	1.216–1.812	< 0.001
Hypertension	0.512	38.642	1.668	1.382–2.015	< 0.001

A weighted prediction model for LPRD was constructed based on the β coefficients from the multivariate logistic regression model. The score for each risk factor was calculated as the β coefficient multiplied by 10 and rounded to the nearest integer, yielding a total score ranging from 0 to 22 points (Table 4). The model demonstrated good calibration, with a non-significant Hosmer-Lemeshow goodness-of-fit test (*P* = 0.324) and a Brier score of 0.142. The diagnostic efficacy of the model at different score thresholds is shown in Table 5. As the score threshold increased, the sensitivity of the model gradually decreased, while the specificity gradually increased. The Youden index reached its maximum value (0.615) when the score threshold was 9.5 points, at which point the model had a sensitivity of 0.723 and a specificity of 0.892. This suggests that this threshold is the optimal diagnostic cutoff point for the model, achieving a good balance between sensitivity and specificity.

**Table 4:** Weighted scoring system for the LPRD prediction model.

Risk factor	β coefficient	Weighted score^*^
Age ≥ 65 years	0.586	6
BMI ≥ 25 kg/m^2^	0.632	6
Smoking history	0.425	4
Preference for strong tea	0.498	5
Abnormal LES function	0.865	9
History of laryngeal surgery	0.396	4
Hypertension	0.512	5
Total score range		0–22

^*^Weighted score = β × 10, rounded to the nearest integer.

**Table 5:** Sensitivity and specificity of the prediction model for LPRD in outpatients with laryngeal diseases (at different score thresholds).

Total score	Sensitivity	Specificity	Youden index
1.0	1.000	0.000	0.000
2.0	0.986	0.125	0.111
3.0	0.958	0.268	0.226
4.0	0.912	0.436	0.348
5.0	0.856	0.624	0.480
6.0	0.789	0.795	0.584
7.0	0.715	0.886	0.601
8.0	0.623	0.932	0.555
9.0	0.486	0.968	0.454
9.5	0.723	0.892	0.615
10.0	0.325	0.985	0.310
11.0	0.158	0.996	0.154
12.0	0.000	1.000	0.000

The scoring criteria of the prediction model refer to the 7 risk factors included in Table 4, with 2 points assigned to each risk factor. The total score ranges from 0 to 14 points (Age ≥ 65y = 2 points, BMI ≥ 25 kg/m^2^ = 2 points, Smoking history = 2 points, Preference for strong tea = 2 points, Abnormal LES function = 2 points, History of laryngeal surgery = 2 points, Hypertension = 2 points).

**Figure 2:**
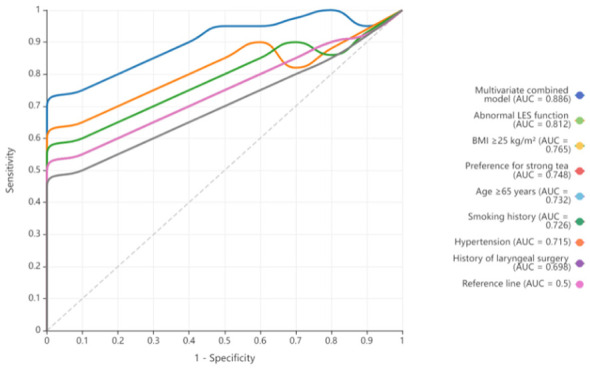
ROC curves of single factors and multivariate combined prediction model for LPRD in outpatients with laryngeal diseases. Area under the curve (AUC) values are labeled next to each curve; the dashed line represents the reference line (AUC = 0.5). The multivariate combined model was constructed based on 7 independent risk factors identified in Table 4 (age ≥ 65 years, BMI ≥ 25kg/m^2^, smoking history, preference for strong tea, abnormal LES function, history of laryngeal surgery, hypertension).

The results of ROC curve analysis for single risk factors and the multivariate combined prediction model (Table 6; Figure 2) showed that all included single risk factors had certain diagnostic value for LPRD (all *P* < 0.001). Among them, abnormal LES function had the highest area under the curve (AUC = 0.812, 95% CI: 0.785–0.839), followed by BMI ≥ 25 kg/m^2^ (AUC = 0.765, 95% CI: 0.736–0.794) and preference for strong tea (AUC = 0.748, 95% CI: 0.719–0.777), while the history of laryngeal surgery had a relatively lower AUC (0.698, 95% CI: 0.668–0.728). The AUC of the multivariate combined prediction model (0.891, 95% CI: 0.870–0.912) was significantly higher than that of all single risk factors, and its ROC curve (Figure 2) was clearly above the curves of each single factor. This indicates that the combined model has superior diagnostic efficacy for LPRD in outpatients with laryngeal diseases and higher clinical application value.

**Table 6:** Diagnostic efficacy of single risk factors and multivariate combined prediction model for laryngopharyngeal reflux disease in outpatients with laryngeal diseases.

Diagnostic indicator	Area under the curve (AUC)	95% confidence interval (CI)	*P* value
Abnormal LES function	0.812	0.785–0.839	< 0.001
BMI ≥ 25kg/m^2^	0.765	0.736–0.794	< 0.001
Preference for strong tea	0.748	0.719–0.777	< 0.001
Age ≥65 years	0.732	0.703–0.761	< 0.001
Smoking history	0.726	0.697–0.755	< 0.001
Hypertension	0.715	0.686–0.744	< 0.001
History of laryngeal surgery	0.698	0.668–0.728	< 0.001
Multivariate weighted prediction model	0.891	0.870–0.912	< 0.001

## Discussion

This study reveals that the incidence of LPRD in patients with laryngeal diseases is closely associated with baseline characteristics such as age, BMI, and gender, which is consistent with the findings of multiple clinical studies at home and abroad. Elderly patients are identified as a high-risk group for LPRD, and the underlying mechanism may be directly related to age-related degenerative changes in physiological functions: with increasing age, the elasticity of the lower esophageal sphincter (LES) smooth muscle naturally decreases, leading to impaired anti-reflux barrier function. Meanwhile, the rate of gastrointestinal peristalsis slows down and salivary secretion reduces, further decreasing the clearance capacity of refluxate and resulting in prolonged exposure of the laryngopharyngeal mucosa to reflux stimulation (18). The strong correlation between obesity and LPRD has been widely confirmed in previous studies (19, 20), and this research further validates this conclusion. The core mechanism not only involves the destruction of LES tone caused by increased intra-abdominal pressure but also may be related to systemic low-grade inflammation induced by inflammatory factors (e.g., TNF-α, IL-6) secreted by adipose tissue (21). This inflammatory response can enhance the sensitivity of the laryngopharyngeal mucosa, making patients exhibit obvious symptoms in response to mild reflux stimulation. Regarding gender differences, the higher prevalence of LPRD in males than in females is closely associated with the higher incidence of unhealthy lifestyle habits such as smoking and alcohol consumption in the male population (22). Additionally, studies (23, 24) have indicated that the regulatory effect of hormone levels on LES function may differ between males and females, and this dimension of the mechanism deserves further in-depth exploration.

### Correlation between lifestyle habits and LPRD and their mechanisms of action

Smoking, preference for strong tea, and spicy food are independent risk factors for LPRD, and their pathogenic mechanisms are characterized by multiple links and targets. The impact of smoking on reflux diseases has been fully confirmed: nicotine in tobacco can directly act on the acetylcholine receptors of LES smooth muscle, leading to sphincter relaxation (25, 26). Meanwhile, nicotine can inhibit the repair capacity of mucosal epithelial cells and reduce the defense threshold of the laryngopharyngeal mucosa, which is consistent with the significant impact of smoking on LPRD observed in this study. Notably, studies (27, 28) have suggested that there may be a synergistic effect between smoking and reflux—smoking not only increases the probability of reflux but also aggravates the degree of mucosal damage caused by reflux, forming a vicious cycle of “damage-sensitivity-exacerbated reflux.” The pathogenic effects of strong tea and spicy food are mainly achieved through dual pathways: caffeine and theophylline in strong tea can stimulate parietal cells to secrete gastric acid, increasing gastric acidity and enhancing the corrosiveness of refluxate; while capsaicin and other components in spicy food can directly destroy the tight junctions of epithelial cells in the esophagus and laryngopharyngeal mucosa, impairing the mucosal barrier function (29, 30). Both factors together exacerbate reflux-related symptoms. This finding further highlights the fundamental role of lifestyle intervention in the prevention and treatment of LPRD.

This study is the first to systematically analyze the association between multiple comorbidities and LPRD in a specialized population of patients with laryngeal diseases, finding that coronary heart disease, chronic obstructive pulmonary disease (COPD), diabetes mellitus, and other comorbidities are associated with an increased risk of LPRD, providing important references for clinical comprehensive management. The association mechanism between comorbidities and LPRD is complex: on one hand, therapeutic drugs for some underlying diseases may indirectly affect reflux risk—for example, nitrates commonly used in patients with coronary heart disease can relax smooth muscle, including the LES, thereby reducing its anti-reflux capacity (31); on the other hand, the pathophysiological changes of the underlying diseases themselves may form a mutually reinforcing relationship with LPRD (32). For instance, the periodic increase in intra-abdominal pressure caused by long-term coughing in COPD patients can repeatedly impact the LES barrier, while gastrointestinal neuropathy in diabetic patients can delay gastric emptying, increasing gastric content retention and creating conditions for reflux (33, 34). In addition, chronic laryngopharyngeal discomfort caused by LPRD may increase the psychological burden on patients with underlying diseases, affecting their treatment compliance and further leading to poor control of the underlying diseases, forming a vicious cycle of multi-system diseases (35, 36). This finding suggests that clinicians should establish a multidisciplinary thinking when managing patients with laryngeal diseases combined with multiple underlying diseases and incorporate LPRD screening into the routine evaluation system.

As the primary independent risk factor for LPRD, the core role of abnormal LES function has been fully validated in this study, which is highly consistent with the pathophysiological nature of LPRD. As the first physiological barrier against esophageal reflux, the functional integrity of the LES is crucial for maintaining the esophagogastric motility balance. Previous studies have confirmed that decreased LES pressure and increased relaxation frequency are the direct pathological bases for gastroesophageal reflux. Compared with gastroesophageal reflux disease (GERD), reflux in LPRD patients is more likely to involve the laryngopharynx, which may be related to the characteristics of abnormal LES function in LPRD patients—some LPRD patients may only exhibit transient LES relaxation rather than persistent pressure reduction. This intermittent relaxation is more likely to cause refluxate to break through the upper esophageal sphincter and directly stimulate the laryngopharyngeal mucosa. The laryngopharyngeal mucosa is more fragile than the esophageal mucosa and lacks sufficient mucus protection. Long-term reflux stimulation can induce a series of pathological changes such as mucosal congestion, edema, and follicular hyperplasia, ultimately manifesting as typical symptoms such as foreign body sensation and hoarseness. The results of this study further support the inclusion of LES function assessment as a key link in LPRD diagnosis, providing a clear direction for etiological targeted therapy.

The LPRD prediction model constructed based on multiple independent risk factors exhibits excellent diagnostic efficacy. Its advantage lies in breaking through the limitations of single-index diagnosis and realizing the integrated evaluation of multi-dimensional risk factors. The model is proposed as a prescreening tool to identify high-risk patients who would benefit from further objective testing (such as impedance-pH monitoring), rather than as a replacement for traditional diagnostic methods. It offers the advantages of simplicity, low cost, and non-invasiveness, making it suitable for rapid screening in outpatient settings and primary medical institutions. However, it is important to acknowledge that the model cannot distinguish between acid and non-acid reflux, nor does it capture laryngeal hypersensitivity, which may be better characterized by objective testing. Previous studies (3, 37) have attempted to construct LPRD risk prediction models, but most have focused on the general population or GERD patients. The model constructed in this study for the specialized population of laryngeal diseases is more targeted, and its AUC value is significantly higher than that of single risk factors, indicating that the model has higher diagnostic value for the target population. Although obesity and smoking are well-established risk factors for GERD, their role in LPR—particularly in patients with primary laryngeal diseases—remains less well characterized. Our findings confirm their independent contribution in this specific outpatient population, reinforcing the importance of lifestyle interventions in this subgroup. The clinical application of this model can not only improve the early identification rate of LPRD but also provide precise targets for clinical intervention—for high-risk patients, lifestyle intervention or preventive medication can be initiated in advance to reduce the incidence of LPRD or alleviate the severity of symptoms. In the future, multi-center large-sample studies can be conducted to further verify the external validity of the model, and artificial intelligence algorithms can be combined to optimize model parameters and improve its diagnostic accuracy.

Combined with the results of this study and existing clinical evidence, the prevention and treatment of LPRD complicating laryngeal diseases should adopt a full-process comprehensive intervention strategy of “diagnosis-treatment-nursing-management.” At the treatment level, the principle of synergistic treatment of the primary disease and LPRD should be adhered to. On the basis of controlling the symptoms of laryngeal diseases, standardized anti-reflux treatment should be administered. Proton pump inhibitors (PPIs) are the first-line treatment drugs, and sufficient course of treatment should be guaranteed to ensure the effect of gastric acid inhibition. For patients with poor PPI response, prokinetic drugs or mucosal protectants can be combined, and endoscopic or surgical evaluation may be required to optimize the treatment plan when necessary. The core of nursing intervention lies in lifestyle guidance. Individualized nursing plans should be formulated based on the risk factors identified in this study: for obese patients, scientific weight loss plans should be developed; for patients with unhealthy dietary preferences, specific dietary substitution suggestions should be provided; at the same time, health education on the hazards of smoking should be strengthened to assist patients in formulating smoking cessation plans. At the management level, a screening and long-term follow-up system for high-risk groups should be established, incorporating risk factors such as age, BMI, and smoking history into the screening and evaluation system, and conducting regular symptom monitoring and functional assessment for high-risk patients. Training of medical staff should be strengthened to improve their ability to identify atypical symptoms of LPRD and avoid missed diagnosis and misdiagnosis. Continuous health education should be carried out through doctor-patient communication platforms to improve patients' understanding of the disease and treatment compliance, ultimately achieving effective prevention and control of LPRD.

## Limitations

Although this study has obtained valuable findings, several limitations should be acknowledged. First, the study adopted a single-center cross-sectional design, with all participants recruited from a single institution, which may introduce selection bias and limit the generalizability of the findings. Moreover, the cross-sectional nature precludes establishing causal relationships between the identified risk factors and LPRD. Second, objective assessments such as high-resolution esophageal manometry and 24-h pH monitoring were not performed in all patients (HRM was performed in 29.5% of the total cohort, and pH monitoring was used selectively as clinically indicated), which may not only introduce selection bias at the time of enrollment but also affect the accuracy of the experimental results, particularly by underdiagnosing non-acid or weakly acidic reflux. This limitation is a major concern and warrants careful interpretation of the findings. Third, widely used tools such as the Reflux Symptom Index (RSI) and Reflux Finding Score (RFS) were not systematically collected in this retrospective cohort. Fourth, the collection of lifestyle factors (e.g., dietary habits, smoking history) relied on patient self-report, which may be subject to recall bias. Fifth, several potential confounders—including alcohol consumption, hiatal hernia, anxiety/depression, medication use (e.g., calcium channel blockers, nitrates), sleep apnea, and proton pump inhibitor use—were not consistently recorded in the medical records and therefore could not be included in the analysis. Sixth, although we employed a clinical reasoning-based variable selection approach guided by prior literature and pathophysiological plausibility to avoid the risks associated with stepwise regression, this approach may still be subject to investigator bias and warrants validation in independent datasets. Finally, the prediction model was not validated in an external cohort, and long-term follow-up data on model-guided interventions are lacking; thus, its ability to reduce LPRD incidence or improve clinical outcomes remains to be confirmed. Future multi-center prospective studies with standardized diagnostic protocols and comprehensive data collection are needed to address these limitations and further refine the risk assessment system.

## Conclusion

Drawing on real-world data from 1,650 consecutive outpatients with laryngeal disorders, we delineated the epidemiological profile and risk factors for LPRD. The condition was present in 44.7% of the cohort and was associated with a phenotype characterized by advanced age, elevated body mass index, multiple comorbidities, and cumulative lifestyle exposures. Multivariable logistic regression identified hypertension as an independent determinant of LPRD alongside the well-established risk factor of lower esophageal sphincter dysfunction, suggesting a potential gastro-laryngo-cardiovascular axis. A seven-item scoring system derived from these associations achieved an area under the curve of 0.886; at the optimal cut-off of 7 points, specificity was 88.6% with a false-negative rate of 28.5%, providing a practical quantitative triage tool for use in busy otolaryngology clinics.

To facilitate translation into clinical practice, we propose that individuals aged ≥ 65 years, with a body mass index ≥ 25 kg/m^2^, a history of smoking or habitual consumption of strong tea or spicy food, concurrent hypertension, or previous laryngeal surgery undergo routine prescreening using this model. Those with a score ≥7 could proceed directly to 24-h multichannel intraluminal impedance-pH monitoring. A multidisciplinary “digestive–ENT–cardiology” clinic could then deliver a four-pronged intervention—proton pump inhibitor, prokinetic agent, weight reduction, and blood pressure control—while favoring angiotensin receptor blockers over calcium channel blockers to avoid exacerbating reflux. Patient self-management might be enhanced through a WeChat-based “laryngeal reflux diary” that captures dietary intake, voice use, blood pressure readings, and symptom scores, enabling remote clinician review. Mechanistically, animal models investigating the hypertension–LES dysfunction–laryngeal mucosal microinjury axis are needed to clarify how the renin–angiotensin system modulates sphincter tone and pharyngeal epithelial barrier function. Future integration of pharyngeal microbiome profiles, vocal fold vibration parameters, and artificial intelligence-based voice analysis into a continuously updated cloud algorithm could improve detection of atypical LPRD presentations such as silent reflux. Lastly, multicenter randomized controlled trials comparing standard proton pump inhibitor therapy with a combined regimen incorporating aggressive blood pressure control and voice therapy will be essential to establish evidence-based strategies that concurrently reduce LPRD recurrence and cardiovascular events.

## Data Availability

The raw data supporting the conclusions of this article will be made available by the authors, without undue reservation.
